# Forecasting the Short-Term Passenger Flow on High-Speed Railway with Neural Networks

**DOI:** 10.1155/2014/375487

**Published:** 2014-11-04

**Authors:** Mei-Quan Xie, Xia-Miao Li, Wen-Liang Zhou, Yan-Bing Fu

**Affiliations:** School of Traffic and Transportation Engineering, Central South University, Changsha 410075, China

## Abstract

Short-term passenger flow forecasting is an important component of transportation systems. The forecasting result can be applied to support transportation system operation and management such as operation planning and revenue management. In this paper, a divide-and-conquer method based on neural network and origin-destination (OD) matrix estimation is developed to forecast the short-term passenger flow in high-speed railway system. There are three steps in the forecasting method. Firstly, the numbers of passengers who arrive at each station or depart from each station are obtained from historical passenger flow data, which are OD matrices in this paper. Secondly, short-term passenger flow forecasting of the numbers of passengers who arrive at each station or depart from each station based on neural network is realized. At last, the OD matrices in short-term time are obtained with an OD matrix estimation method. The experimental results indicate that the proposed divide-and-conquer method performs well in forecasting the short-term passenger flow on high-speed railway.

## 1. Introduction

Short-term passenger flow forecasting is an important component of transportation systems which can be used to fine-tune travel behaviors, enhance service quality, reduce passenger crowd, and raise revenues of transportation systems. The forecasting results of short-term passenger flow can be applied to support transportation system operation and management such as operation planning, station passenger crowd regulation planning, and revenue management.

As a rapid intercity transportation mode, high-speed railway is developing rapidly in many countries and has become an emerging trend worldwide. In competition with aviation and road infrastructure, high-speed railway shows safer, more convenience, and more efficient performance in terms of land use and energy efficiency. In China, high-speed railway, as an immature transport mode, effectively relieves the high pressure of passenger demands of busy trunk railway lines among the major cities. From the view of economy, high-speed railway is also a high-cost commodity. And the economic principle of allocating investments to high-speed railway is dependent on passenger flows. If the forecasting results of the short-term passenger flow on high-speed railway are known well by the decision maker, the operational cost such as staff and facility cost can be controlled. It is an important issue to support sustainable development for high-speed railway.

The expression forms of passenger flow are varied in railway system. The OD matrix is one form. The number of passengers travelling on a railway line or in a railway network is another, and if you want to get OD matrix, passenger assignment is a right and ordinary choice. In this paper, the former stands for passenger flow. That is to say, forecasting the short-term passenger flow on high-speed railway is to forecast the OD matrices in short-term period.

Theoretically, if every OD pair is forecasted separately and then combined, the OD matrix table of predicted passenger flow can be got. But it is a huge workload. The research motivation of this paper is a novel and time-saving method of short-term passenger flow forecasting based on neural networks. The contributions are as follows: (i) the divide-and-conquer method forecasts the passenger flow between stations, which are great contribution to line planning, especially the stop modes for trains; (ii) it gives a frame to predict the passenger flow in special holiday.

The remainder of this paper is structured as follows. In Section 2 we give a literature overview.  Section 3 describes the short-term passenger flow forecasting problem and discusses the divide-and-conquer method in detail. In Section 4 we design a numerical example and do some reasons analysis. Finally, we draw some conclusions in Section 5.

## 2. Literature Review

There is a rich list of publications on short-term transportation forecasting. The most common approaches to cope with short-term forecasting problems are extrapolation. Many different model prototypes have been applied, and they can be divided into three categories generally: parametric and nonparametric techniques [1, 2] and hybrid ones. Parametric techniques and nonparametric techniques refer to the functional dependency assumed between independent variables and the dependent variable [3].

For the parametric techniques, several methods have been used to forecast transportation demand, and autoregressive integrated moving average (ARIMA) [4] is used mostly. With the characteristics of seasonality and trends, seasonal ARIMA has been applied to forecast traffic flow [5, 6]. However, the applications of ARIMA and seasonal ARIMA models are limited because they assume linear relationships among time-lagged variables so that they may not capture the structure of nonlinear relationships [7].

In the nonparametric techniques, neural network [8], nonparametric regression [1], and Gaussian maximum likelihood [9] have been applied to forecast transportation demand. Among these methods, neural networks have been frequently adopted as the modeling approach because they possess the characteristics of adaptability, nonlinearity, and arbitrary function mapping capability [7]. The passenger flows on high-speed railway have some nonlinear characteristics, so the method of neural networks, plus optimized and metaoptimized one [10–13], is used by scholars. And the method is also used in this paper.

For hybrid techniques, there are lots of publications on traffic flow forecasting, for instance, a hybrid model that combines both wavelets analysis and neural network [14], empirical mode decomposition, and neural networks [3]. Recently, Jiang et al. [15] proposed a hybrid approach combining ensemble empirical mode decomposition and gray support vector machine to forecast the short-term high-speed rail demand, which is demonstrated with three typical OD pairs along the Wuhan-Guangzhou high-speed railway in China.

For the ordinary urban networks, estimating OD matrix by road traffic flows mainly includes several steps as follows: checking and measuring road flow and obtaining a priori information; procuring road network characters and traffic assignment matrix; estimating OD matrix according to special estimate models. In the above steps, the main factors influencing the precision of OD estimation are the accuracy of the estimate model, reliability of a priori information, accuracy of road flow checked and measured, and the rationality of the traffic assignment method. In addition, the solution to the model is also a problem worthy of discussion and the feasibility, simplicity, and convenience of the solution influence the applicability of the model.

On railway operation, there are also some publishers to tackle with the problem of OD estimation [16], but the structured approach cannot meet the requirement of real-time performance and prediction precision. So a new OD estimation method is discussed in Section 3.

## 3. Methods

### 3.1. Problem Definition

The term “OD matrix” means the number of passengers of each OD pair on the railway line, not for the trains, just as shown in Table 1.

There are some variables that should be explained in Table 1.

 
*S*_*n*_ is Station *n*. There are *n* stations on the railway line.
 
*X*_*ij*_ is the numbers of passengers who depart from Station *i* and arrive at Station *j*; that is to say, travel from Station *i* to Station *j* by the train. While *i* = *j*, *X*_*ij*_ = 0.
 
*X*_*i*_ is the numbers of passengers who arrive at Station *i* and depart from all the stations but Station *i* on the railway line.
 
*Y*_*j*_ is the numbers of passengers who arrive at all the stations but Station *j* on the railway line and depart from Station *j*.

And we can draw up some equations from Table 1 as follows:

(1) Xj=∑i=1nXij,

(2) Yi=∑j=1nXij,

(3) ∑j=1nXj=∑i=1nYi.

Equation (3) shows that the number of passengers who depart from the different origin stations is equal to the number of passengers who arrive at the destination stations in the railway system.

Theoretically, there is a superposition method to predict the short-term passenger flow. That is to say, if every OD pair is forecasted separately and then combined, the OD matrix table of predicted passenger flow can be got. If there are *n* stations in the railway network, there will be *n*∗(*n* − 1) OD pairs. So to that extent, the amount of work is very huge. So a divide-and-conquer and timesaving method is proposed in this paper.

### 3.2. Forecasting Method

**Figure 1:**
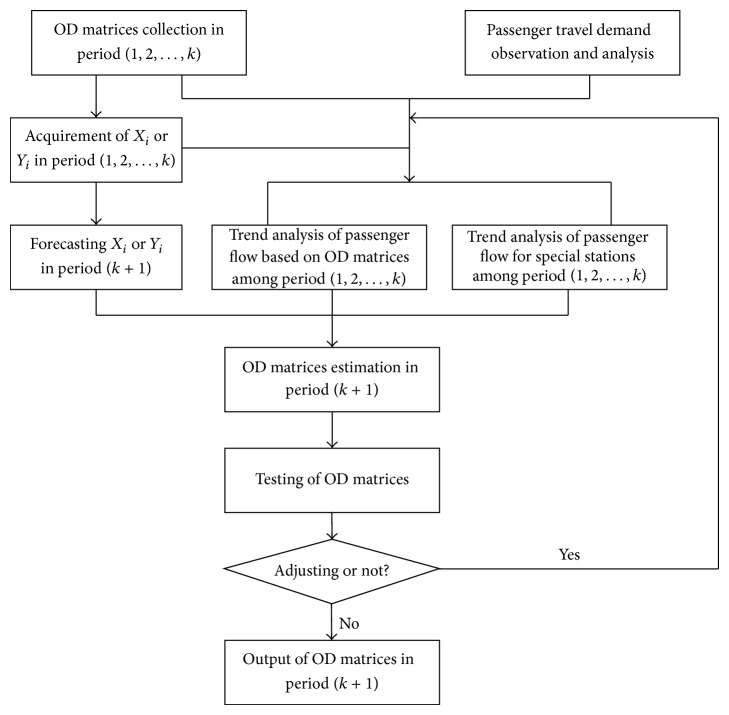
The forecasting process of short-term passenger flows.

The divide-and-conquer method has three steps, and the historical and present OD matrices and passenger travel demand are the basis, just as shown in Figure 1. The method will be introduced in detail as follows.

**Step 1 (acquirement of *X*_*i*_ or *Y*_*i*_ in period (1,…, *k*)).**

In order to get the short-term passenger flow, we must get the historical and present OD matrices data which would be called OD matrix table in period from 1 to *k*, and the bigger the number is, the closer the data is to the forecasting period.

The data *X*_*i*_ and *Y*_*i*_ are easy to get with (1) and (2).

**Step 2 (forecasting *X*_*i*_ or *Y*_*i*_ in period (*k* + 1) based on neural network).**

**Figure 2:**
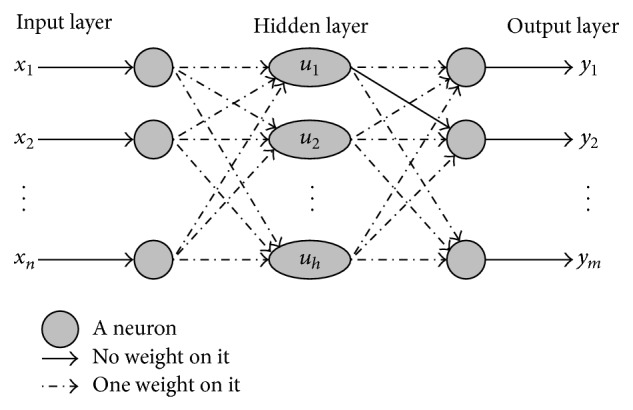
Multilayer feed forward neural network structure.

With the continuing increase in computing power and availability of data, there has been a growing interest in the use of artificial neural networks for forecasting purposes. And the most commonly used form for forecasting is the feed forward multilayer perception. It is a forward connected network which usually has three layers named input layer, hidden layer, and output layer, as shown in Figure 2.

Input layer is used for receiving information from external inputs. The number of neurons in input layer depends on the number of input features such as holiday data, weekend data, daily data, and monthly data for passenger flow on high-speed railway. Hidden layer can be seen as a feature extractor. It mixes information from input layer and generates new features for network learning. The number of neurons in hidden layer is case by case. Output layer generates forecasts and propagates errors for parameter estimation. The number of neurons in output layer depends on how many lead-time forecasts are requested.

Further, in Figure 2, each broken line between different layers has one weight on it and represents a parameter. And the adjustment of these weights is done by backpropagation algorithm, so BP neural network turns into a common forecasting method and it will be used in this paper to forecast *X*_*i*_ or *Y*_*i*_ in period (*k* + 1). That is to say, the values of *X*_*i*_ or *Y*_*i*_ in period (1,…, *k*) are input data and *X*_*i*_ or *Y*_*i*_ in period (*k* + 1) are output data.

Theoretically, the hidden layer can be more than one layer, and the more the layers are, the higher the precision of forecasting value is. But if there are more enough neurons in hidden layer for single-hidden layer neural network, the precision of forecasting value will also be higher. The single-hidden layer neural network is used in theory research and practical application frequently. Its functional form is

(4) yi=∑j=1Hwij2σ∑k=1Nwjk1xk+θj1+θi2 i=1,2,…,M,

where *H* is the number of neurons in hidden layer; *N* is the number of neurons in input layer; *M* is the number of neurons in output layer; *y*_*i*_ is output value of *i*th neuron; *x*_*k*_ is input value of *k*th neuron; *w*_*jk*_^1^ is the connection weight between *j*th neuron in hidden layer and *k*th neuron in input layer; *w*_*ij*_^2^ is the connection weight between *j*th neuron in hidden layer and *i*th neuron in output layer; *σ*(·) is activation function of neurons; *θ*_*j*_^1^ is the threshold value of *j*th neuron in hidden layer; *θ*_*i*_^2^ is the threshold value of *i*th neuron in output layer.

Above all, single-hidden layer BP neural network is applied in this paper.

**Step 3 (OD estimation).**

The method of passenger flow forecasting based on OD backward induction gives out a way to get the OD matrices [16], but the structured approach cannot meet the operation requirement in real-time performance and prediction precision. If we have enough historical data in the process of railway operation, the way of inferring the OD matrix is feasible [17]. This way is called OD estimation in this paper.

In order to describe the problem commodiously, *r*_*ij*_ is defined.

*r*_*ij*_ is a ratio of passenger flow in the form of OD (Table 2). It can be described as follows:

(5) rij=xijYi.

While *i* = *j*, *r*_*ij*_ = 0; *r*_*ij*_′ = 0. And ∑_*j*=1_^*n*^ *r*_*ij*_ = 1; ∑_*i*=1_^*n*^ *r*_*ij*_′ = 1.

In every period, the value of *r*_*ij*_ is different, so we define *r*_*ij*_^(*t*)^ as the ratio *r*_*ij*_ in period (*t*). If we get the values of *Y*_*i*_ and *r*_*ij*_ (or *X*_*i*_ and *r*_*ij*_′) in period (*k* + 1), the OD matrix in period (*k* + 1) will be obtained easily.

The value of *Y*_*i*_ in period (*k* + 1) will be realized in Step 2. So the value of *r*_*ij*_ in period (*k* + 1) is critical. In the same condition (weather, holiday, weekend, and so on), there will be some trend among these values of *r*_*ij*_  in successive period. For example, the values of *r*_*ij*_  in period (*t*) and period (*t* + 1) have some stable transitive relation. So we can let the *r*_*ij*_^(*k*)^ replace *r*_*ij*_^(*k*+1)^ approximatively. Of course, the replacement should be tested and adjusted, just as shown in Figure 1.

Besides, there are some special issues to put in words. At first, the OD matrices among the same type of holiday (3-day holiday, 7-day holiday, the Spring Festival, etc.) have some special trends, called holiday trends for short in this paper and which Jiang et al. [15] studied lately. The short-term passenger flow forecasting in holiday is a special issue which combines the holiday trends and conventional forecasting program and so does the weekend. In this paper, we will not study the holiday trends, so the data referring to holiday is left out in the next part. Secondly, the part “adjusting or not” in Figure 1 should be handled based on some rules, especially for passenger flow forecasting in holidays.

## 4. Numerical Example and Reasons Analysis

Assume a high-speed railway line which is put into operation in short time with 15 stations. There are 3 stations in big city, namely,* S*_1_,* S*_10_, and* S*_15_, with more passengers. We got the OD matrices for the historical passenger flow on the line in successive 10 months. A month stands for a period. The detailed OD matrices are left out in this paper, but the values of *Y*_*i*_ in period (1,…, 10) have been organized in Table 3. For simplicity, some special data in special days are not taken into consideration, such as holiday and weekend. So *Y*_*i*_  (*i* = 1,…, 15) is an average number for daily data in the *t*th  (*t* = 1,…, 10) month.

Samples used for training are described as follows. The data in period (1,2, 3) is used as the first input vector, and the data in period (4) is the corresponding output vector. The data in period (2,3, 4) is used as the second input vector, and the data in period (5) is the corresponding output vector. The rest may be deduced by analogy. Then we have got 7 training samples.

**Figure 3:**
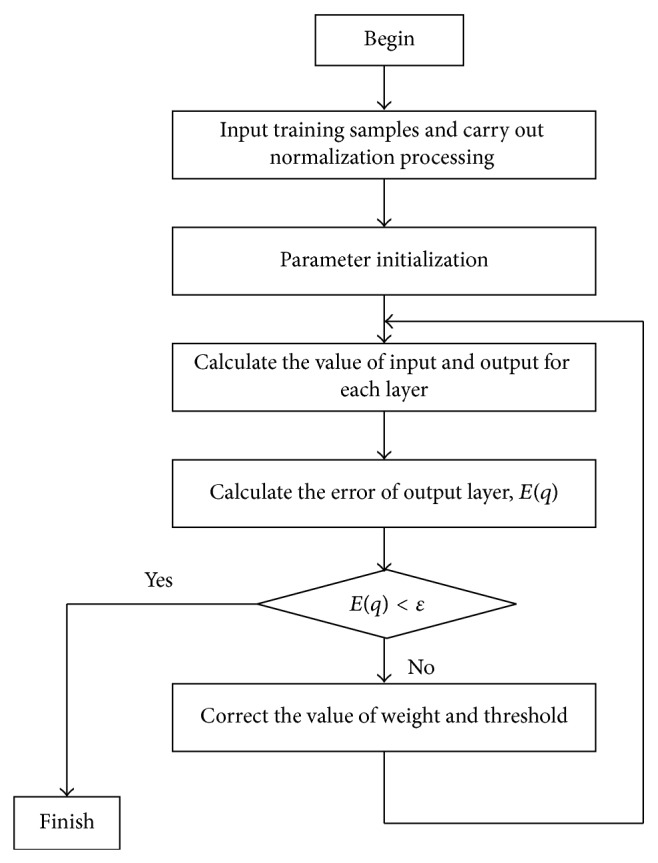
The running process of BP networks.

Before the forecasting work, the running process of BP networks is described as shown in Figure 3.

In this paper, training samples are the data of *Y*_*i*_ in period (1,…, 9). Parameter initialization includes the initialization of maximum training time, training goal, neurons of hide layers, and so on. The number of neurons of hide layers is 50, maximum training time is 1500, and training goal is 0.01 in this numerical example.

By virtue of the platform of MATLAB, program for neural network is run. Then the forecasting result is shown in Table 4. And *E*(*q*) is the error of the output layer. The values of *E*(*q*) are all smaller than 0.01, so the forecasting results are acceptable.

After data mining from passenger ticket system, the OD matrix can be obtained. Further, the ratio of passenger flow in period (10) is just shown in Table 5. And OD matrix of the passenger flow in period (11) can be calculated with (5) or (6), as shown in Table 6.

So far, we have got the passenger flow in period (11) as shown in Table 6. From Table 6, we can get some conclusions as follows.

1. The passenger flow between* S*_1_ and* S*_10_,* S*_1_ and* S*_15_, and* S*_10_ and* S*_15_ is bigger than others.
2. The passenger flow between the same two stations is not much difference. For instance, the passenger flow from* S*_1_ to* S*_2_ is 429 in period (11), and the number is 469 from* S*_2_ to* S*_1_.

The conclusions are very close to the real operation.

The results of short-term passenger flow forecasting can provide useful information for decision maker of high-speed railway systems. With the results of short-term passenger flow forecasting, decision makers can appropriately adjust the operation plans, activate the station passenger crowd regulation plan, and adjust fares. The operation plans can be slightly modified based on the fluctuation of passenger flow to ensure that the required service level of the high-speed railway systems can be met. The minor modifications can be made in the operation plans such as train service plan, train schedule, timetable, train-set circulation, and crew schedule.

In this paper, single-hidden layer BP neural network is used to forecast the numbers of passengers who arrive at each station or depart from each station in period (*k* + 1). Compared with the numerical example, the passenger flows are gradually increasing from month to month, and the forecasting results are bigger than the data in period (*k*). As for a line which is put into operation lately, the attracted passengers will be gradually getting more until it gets stable. So the forecasting results are in line with reality. At the same time, the error in output layer is smaller than 0.01, and the forecasting results can be accepted. Then OD estimation method is used to provide OD matrix quickly; it is practical and timesaving.

## 5. Conclusions and Further Research

An accurate and stable passenger flow casting can be applied to support transportation system operation and management such as operation planning, revenue planning, and facility improvement. This paper proposes a divide-and-conquer method based on neural network for short-term passenger flow with three steps. In the first step, the numbers of passengers who arrive at each station or depart from each station, namely, *Y*_*i*_ or *X*_*j*_, in period (1,…, *k*) are extracted from the short-term passenger flow, OD matrix in this paper, which can be mined from passenger ticket system. And as to special days such as National Day, there are some trends among the same type of holiday on passenger flow, so part of data is not taken into consideration in this paper. In the second step, short-term passenger flow forecasting for the numbers of passengers who arrive at each station or depart from each station in period (*k* + 1) based on neural network is realized. In the third step, OD matrix estimation method is used to get the OD matrices in short-term period. The experimental results indicate that the proposed divide-and-conquer method performs well in forecasting the short-term passenger flow on high-speed railway. In particular, the short-term passenger flow forecasting in holiday is a special issue which combines the trends and conventional forecasting program; it is the work to be further studied.

## Figures and Tables

**Table 1:** OD matrix table of passenger flow.

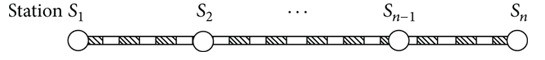					
	Destination				$\overset{n}{\underset{j=1}{\sum }}X_{ij}$
	*S*_1_	*S*_2_	*⋯*	*S*_*n*_	

Origin					
*S*_1_	*X*_11_	*X*_12_	*⋯*	*X*_1*n*_	*Y*_1_
*S*_2_	**X**_21_	*X*_22_	*⋯*	*X*_2*n*_	*Y*_2_
*⋯*	*⋯*	*⋯*	*⋯*	*⋯*	*⋯*
*S*_*n*_	*X*_*n*1_	*X*_*n*2_	*⋯*	*X*_*nn*_	*Y*_*n*_
$\overset{n}{\underset{i=1}{\sum }}X_{ij}$	*X*_1_	*X*_2_	*⋯*	*X*_*n*_	

**Table 2:** OD ratios of passenger flows.

	Destination				$\sum_{j=1}^{n}r_{ij}$
	*S*_1_	*S*_2_	*⋯*	*S*_*n*_	
Origin					
*S*_1_	*r*_11_	*r*_12_	*⋯*	*r*_1*n*_	1
*S*_2_	**r**_21_	*r*_22_	*⋯*	*r*_2*n*_	1
*⋯*	*⋯*	*⋯*	*⋯*	*⋯*	*⋯*
*S*_*n*_	*r*_*n*1_	*r*_*n*2_	*⋯*	*r*_*nn*_	1

**Table 3:** The values of *Y*_*i*_ in period (1,…, 10).

*Y*_*i*_	Period									
	1	2	3	4	5	6	7	8	9	10
*Y*_1_	16089	16477	17514	18025	19038	20441	21001	21227	22406	23077
*Y*_2_	2333	2389	2540	2614	2761	2964	3045	3078	3249	3346
*Y*_3_	844	865	919	946	999	1073	1102	1114	1175	1210
*Y*_4_	2643	2707	2878	2962	3128	3359	3451	3488	3681	3791
*Y*_5_	3892	3986	4237	4360	4605	4945	5080	5135	5420	5582
*Y*_6_	1197	1225	1303	1340	1416	1520	1562	1578	1666	1716
*Y*_7_	3751	3842	4084	4203	4439	4766	4897	4949	5224	5380
*Y*_8_	1237	1267	1346	1386	1463	1572	1614	1632	1722	1773
*Y*_9_	2292	2347	2495	2568	2712	2912	2992	3024	3192	3287
*Y*_10_	12970	13283	14120	14532	15349	16479	16931	17113	18063	18604
*Y*_11_	446	457	485	499	528	566	582	588	620	639
*Y*_12_	2550	2611	2776	2857	3017	3240	3328	3364	3551	3657
*Y*_13_	451	463	492	506	534	574	589	595	629	647
*Y*_14_	499	510	543	559	590	633	650	657	694	714
*Y*_15_	7420	7599	8078	8313	8781	9428	9686	9790	10333	10643

Total	58614	60028	63810	65670	69360	74472	76510	77332	81625	84066

**Table 4:** The forecasting results in period (11).

Station	*Y*_*i*_	*E*(*q*) < 0.01
*S*_1_	23203	0.0096
*S*_2_	3509	0.0028
*S*_3_	1234	0.0029
*S*_4_	3878	0.0062
*S*_5_	5726	0.0040
*S*_6_	1770	0.0044
*S*_7_	5477	0.0031
*S*_8_	1898	0.0035
*S*_9_	3415	0.0058
*S*_10_	19042	0.0036
*S*_11_	664	0.0027
*S*_12_	3736	0.0037
*S*_13_	667	0.009
*S*_14_	749	0.0053
*S*_15_	11015	0.0037

**Table 5:** *r*_*ij*_^(10)^ of passenger flow (%).

O	D														
	*S*_1_	*S*_2_	*S*_3_	*S*_4_	*S*_5_	*S*_6_	*S*_7_	*S*_8_	*S*_9_	*S*_10_	*S*_11_	*S*_12_	*S*_13_	*S*_14_	*S*_15_
*S*_1_	0	1.85	1.65	9.29	6.43	2.26	8.92	2.8	6.86	28.07	1.11	6.07	1.08	1.03	22.58
*S*_2_	13.37	0	0.74	11.27	9.7	5.83	12.77	3.65	4.39	20.84	0.6	4.52	0.89	0.68	10.75
*S*_3_	30.6	1.81	0	28.33	5.67	1.9	5.31	1.82	2.91	11.62	0.58	2.76	0.29	0.22	6.18
*S*_4_	56.36	9.51	8.32	0	4.43	0.97	3.08	1.44	2.02	8.6	0.28	1.04	0.1	0.09	3.76
*S*_5_	28	5.68	1.28	3.45	0	0.77	5.3	1.18	4.94	44.12	0.35	1.72	0.08	0.11	3.02
*S*_6_	34.24	11.91	1.18	2.57	2.57	0	1.54	1.64	3.85	36.65	0.26	0.98	0.1	0.1	2.41
*S*_7_	38.16	6.88	1.05	2.5	5.23	0.58	0	0.36	2.69	33.76	0.38	2.01	0.1	0.11	6.19
*S*_8_	33.47	7.15	0.94	3.28	3.87	1.69	1.49	0	3.92	38.72	0.4	1.69	0.05	0.1	3.23
*S*_9_	49.35	4.46	1.02	2.51	8.68	2	5.18	1.66	0	7.2	0.61	4.22	0.29	0.56	12.26
*S*_10_	36.25	3.53	0.75	1.81	13.13	3.28	10.3	3.48	1.55	0	0.96	7.21	0.39	0.49	16.87
*S*_11_	40.6	4.39	1.24	1.65	3.29	0.55	3.16	0.96	2.74	24.01	0	3.57	0.41	0.54	12.89
*S*_12_	32.23	3.05	0.53	1.27	2.31	0.43	3.05	0.6	3.36	35.18	0.75	0	0.51	0.6	16.13
*S*_13_	43.67	4.35	0.68	0.95	0.82	0.27	1.23	0.14	1.36	11.16	0.54	2.86	0	0.68	31.29
*S*_14_	34.08	2.59	0.49	0.62	0.99	0.25	1.11	0.25	2.59	12.96	0.49	3.34	0.49	0	39.75
*S*_15_	48.74	3.3	0.58	1.36	1.53	0.34	2.95	0.45	3.52	26.81	0.76	5.15	1.8	2.71	0

**Table 6:** Passenger flow in period (11).

	*S*_1_	*S*_2_	*S*_3_	*S*_4_	*S*_5_	*S*_6_	*S*_7_	*S*_8_	*S*_9_	*S*_10_	*S*_11_	*S*_12_	*S*_13_	*S*_14_	*S*_15_
*S*_1_	0	429	383	2156	1492	524	2070	650	1592	6513	258	1408	251	239	5239
*S*_2_	469	0	26	395	340	205	448	128	154	731	21	159	31	24	377
*S*_3_	378	22	0	350	70	23	66	22	36	143	7	34	4	3	76
*S*_4_	2186	369	323	0	172	38	119	56	78	334	11	40	4	3	146
*S*_5_	1603	325	73	198	0	44	303	68	283	2526	20	98	5	6	173
*S*_6_	606	211	21	45	45	0	27	29	68	649	5	17	2	2	43
*S*_7_	2090	377	58	137	286	32	0	20	147	1849	21	110	5	6	339
*S*_8_	635	136	18	62	73	32	28	0	74	735	8	32	1	2	61
*S*_9_	1685	152	35	86	296	68	177	57	0	246	21	144	10	19	419
*S*_10_	6903	672	143	345	2500	625	1961	663	295	0	183	1373	74	93	3212
*S*_11_	270	29	8	11	22	4	21	6	18	159	0	24	3	4	86
*S*_12_	1204	114	20	47	86	16	114	22	126	1314	28	0	19	22	603
*S*_13_	291	29	5	6	5	2	8	1	9	74	4	19	0	5	209
*S*_14_	255	19	4	5	7	2	8	2	19	97	4	25	4	0	298
*S*_15_	5369	363	64	150	169	37	325	50	388	2953	84	567	198	299	0
